# Multimorbidity in Incident Heart Failure: Characterisation and Impact on 1-Year Outcomes

**DOI:** 10.3390/jcm13133979

**Published:** 2024-07-08

**Authors:** Anyuli Gracia Gutiérrez, Aida Moreno-Juste, Clara Laguna-Berna, Alejandro Santos-Mejías, Beatriz Poblador-Plou, Antonio Gimeno-Miguel, Fernando J. Ruiz Laiglesia

**Affiliations:** 1 Internal Medicine Service, Defense General Hospital, Vía Ibérica 1, ES-50009 Zaragoza, Spain; 2 Research Group on Heart Failure, IIS Aragón, Paseo de Isabel la Católica 1-3, ES-50009 Zaragoza, Spain; fruizl@unizar.es; 3 EpiChron Research Group, Aragon Health Sciences Institute (IACS), IIS Aragón, Miguel Servet University Hospital, Paseo de Isabel la Católica 1-3, ES-50009 Zaragoza, Spain; aidamorenoj@gmail.com (A.M.-J.); clagunab.iacs@aragon.es (C.L.-B.); asantosm@ext.aragon.es (A.S.-M.); bpoblador.iacs@aragon.es (B.P.-P.); agimenomi.iacs@aragon.es (A.G.-M.); 4 Research Network on Chronicity, Primary Care and Health Promotion (RICAPPS), ISCIII, ES-28029 Madrid, Spain; 5 San Pablo Primary Care Health Centre, Aragon Health Service (SALUD), de los Aguadores Street 7, ES-50003 Zaragoza, Spain; 6 Internal Medicine Service, Lozano Blesa University Hospital, de San Juan Bosco Street 15, ES-50009 Zaragoza, Spain

**Keywords:** heart failure, comorbidity, multimorbidity, co-existing conditions, mortality, hospital admission

## Abstract

**Background/Objectives**: Heart failure (HF) is usually accompanied by other comorbidities, which, altogether, have a major impact on patients and healthcare systems. Our aim was to analyse the demographic and clinical characteristics of incident HF patients and the effect of comorbidities on one-year health outcomes. **Methods**: This was an observational, retrospective, population-based study of incident HF patients between 2014 and 2018 in the EpiChron Cohort, Spain. The included population contained all primary and hospital care patients with a diagnosis of HF. All chronic diseases in their electronic health records were pooled into three comorbidity clusters (cardiovascular, mental, other physical). These comorbidity groups and the health outcomes were analysed until 31 December 2018. A descriptive analysis was performed. Cox regression models and survival curves were calculated to determine the hazard risk (HR) of all-cause mortality, all-cause and HF-related hospital admissions, hospital readmissions, and emergency room visits for each comorbidity group. **Results**: In total, 13,062 incident HF patients were identified (mean age = 82.0 years; 54.8% women; 93.7% multimorbid; mean of 4.52 ± 2.06 chronic diseases). After one-year follow-up, there were 3316 deaths (25.3%) and 4630 all-cause hospitalisations (35.4%). After adjusting by gender, age, and inpatient/outpatient status, the mental cluster was associated (HR; 95% confidence interval) with a higher HR of death (1.08; 1.01–1.16) and all-cause hospitalisation (1.09; 1.02–1.16). **Conclusions**: Cardiovascular comorbidities are the most common and studied ones in HF patients; however, they are not the most strongly associated with negative impacts on health outcomes in these patients. Our findings suggest the importance of a holistic and integral approach in the care of HF patients and the need to take into account the entire spectrum of comorbidities for improving HF management in clinical practice.

## 1. Introduction

Heart failure (HF) is one of the leading causes of morbidity in the older population, accounting for a significant proportion of hospitalisations and healthcare costs worldwide [1]. The prognosis and survival rates of this condition have improved over time due in part to innovation in diagnostic procedures and therapeutic advances, and HF can now be considered a chronic disease. Rarely appearing in isolation, HF is usually accompanied by the presence of other chronic diseases or comorbidities that, along with other clinical and socioeconomic factors, play an important role in the evolution and prognosis of HF [2].

Multimorbidity, defined as the coexistence of two or more chronic conditions in the same individual, occurs in a large portion of the general population, especially in the oldest old [3]. In Spain, multimorbidity affects approximately four in ten people in the general population, but its prevalence can reach nearly 85% in the 75–89 age band [4]. This high prevalence is likely a consequence of the growing incidence of chronic diseases and the ageing of the population. Multimorbidity comprises a challenge for patients, health systems, and health professionals, mainly due to its association with worse health outcomes, increased risk of mortality, reduced quality of life, and inadequate use of health services [5,6]. Multimorbidity is now the norm rather than the exception in clinical practice and requires a paradigm shift from disease-centred to person-centred care that prioritises the integral and holistic view and care of chronic patients [7].

The high burden of multimorbidity shown by patients with HF comprises not only cardiovascular comorbidities but also non-cardiovascular ones. Multimorbidity has been shown to affect up to 98% of HF patients, who can present an average of nearly seven additional comorbidities [8]. Nevertheless, the clinical consequences of multimorbidity in patients with HF are still unknown, being a current topic of research [2]. Overall, HF patients represent a complex target group with different phenotypes depending on sex, age, comorbidities, physiopathological factors, and prognosis, all of which hamper the clinical management of patients and potentially lead to negative health outcomes. Increased disease burden and severity and risk of hospitalisation and death have indeed been reported in HF patients with non-cardiovascular comorbidities [9]. Moreover, HF has been associated with psychological pathologies like depression, which show prevalence rates of two to three times higher than expected in the general population. These psychological pathologies have been associated with a worse prognosis in terms of mortality, hospitalisation, and functional limitations [10,11].

Although the prevalence of comorbidities in HF patients and their influence on health outcomes have been analysed in a number of studies, most of them focused on prevalent cases of HF rather than incident cases, which have been less studied in this regard [9]. In our opinion, characterisation and analysis of the impact of types of comorbid conditions on hospitalisation and death in patients with incident HF during the first year after diagnosis could help us ascertain which baseline chronic comorbidities have a greater short-term effect on health outcomes, which could be useful for the development of tailored patient management plans that take into account the most relevant comorbidities. This population-based study aimed to describe the demographic and clinical characteristics of patients with incident HF and analyse how their characteristics and comorbidities modulate mortality and health outcomes during the first year of follow-up after diagnosis.

## 2. Materials and Methods

### 2.1. Design and Study Population

We performed an observational study based on the EpiChron Cohort, which links demographic, clinical, pharmacological, and health outcome information of all users of the public health system in the Spanish region of Aragón. This cohort was created in 2011, comprising 1.25 million people (95% of the total population of Aragón). A description of the cohort profile and its data sources can be found elsewhere [12].

For this study, all patients of all ages diagnosed with congestive heart failure in primary or hospital care for the first time between 1 January 2014 and 31 December 2018 were selected (i.e., incident cases in this period). The reason for recruiting incident cases over five years was to obtain a larger sample size to increase the power of this study.

### 2.2. Ethical Considerations

This investigation was carried out in accordance with the principles outlined in the Declaration of Helsinki. The Clinical Research Ethics Committee of Aragón (CEICA) approved the protocol for this research (PI18/082). The requirement for informed consent from the study subjects was waived by the CEICA due to the retrospective study design and the anonymisation of the data.

### 2.3. Study Variables

For each patient, their sociodemographic, clinical, and health outcome characteristics were considered for the analysis. We analysed five sociodemographic variables: gender, age, migration status (native vs. immigrant), residency area (urban vs. rural), and deprivation index of the area (i.e., an aggregated socioeconomic indicator of the area codified in four quartiles from low—Q1—to high—Q4—deprivation) [13].

Regarding clinical variables, we measured the prevalence of specific comorbidities registered in patient’s electronic health records by healthcare professionals, the number of drugs dispensed (those with three or more dispensations during the one-year follow-up), and the prevalence of HF-indicated drugs, according to HF guidelines and recommendations for the years when this study was conducted (2014–2018) [14]. Drugs were analysed using the Anatomical Therapeutic Chemical (ATC) classification system at the third level [15]. We analysed comorbidities with a prevalence higher than 5%, those clinically relevant based on a previous study of HF multimorbidity [8], and the comorbidities prioritised by the Spanish Minister of Health in the national strategy for tackling chronicity [16]. The diagnoses were originally coded using the International Classification of Primary Care, Second Edition (ICPC-2) or the International Classification of Diseases, Ninth Edition (ICD-9), depending on the data source used (primary or hospital care, respectively). In order to reduce the number of different diagnosis codes, we used the Agency for Healthcare Research and Quality’s Clinical Classification Software (CCS, version ICD-9-CM, Agency for Healthcare Research and Quality, Rockville, MD, USA) to group the original codes. The CCS diagnostic categories were considered as chronic if they met any of the following criteria: (i) require ongoing interventions using special medical products, services, and equipment and (ii) entail limitations in self-care, social interactions, and independent living [17]. Each chronic disease was also grouped into one of three comorbidity groups depending on their nature: cardiovascular (CV), other physical, and mental [9]. Correspondingly, each comorbidity group included the following diseases. Firstly, the CV group was composed of arterial hypertension (AHT), disorders of lipid metabolism, diabetes mellitus (DM), obesity, acute myocardial infarction (AMI), cardiac dysrhythmias, and cardiac valvular disorders. Secondly, the other physical conditions group included chronic kidney disease, thyroid disorders, chronic obstructive pulmonary disease (COPD) and bronchiectasis, neoplasms, acute cerebrovascular disease (ACD), deficiency and other anaemia, asthma, arthritis, and osteoporosis. Lastly, the mental comorbidity group included depression and mood disorders, delirium, dementia, and amnestic and other cognitive disorders. Multimorbidity was defined as the presence of two or more chronic diseases, and polypharmacy was defined as the dispensation of five or more drugs. Additionally, we analysed whether the HF diagnosis was conducted during a hospitalisation admission or not (inpatient vs. outpatient).

Regarding health outcomes, our research focused on a broad range of health outcomes, including all-cause mortality, hospital admissions due to HF, all-cause hospital admissions, hospital readmissions, and number of emergency visits during the one-year follow-up after HF diagnosis. In order to analyse the aforementioned health outcomes, patients were followed from HF diagnosis until 31 December 2018, the date of the health outcome of interest, or the date of withdrawal from the cohort, whichever occurred first.

### 2.4. Statistical Analyses

Firstly, a descriptive analysis of the study population stratified by gender was performed. Continuous data were expressed as means and standard deviations and categorical data were expressed as frequencies and proportions. For comparisons between sexes, the T-test and chi-squared test were used depending on the nature of each variable; the results were expressed with their 95% confidence intervals (95% CI).

Secondly, Cox regression models and survival curves were calculated to determine the association between each comorbidity group (independent variable) and the health outcomes analysed (dependent variable). All the analyses were performed globally, stratified by gender, and adjusted by age. Afterward, all-cause mortality and the rest of the hospitalisation outcomes were calculated by gender for each chronic disease, adjusting by age and inpatient or outpatient status. As a sensitivity analysis, the models were also stratified by age group. The significance level was set to *p* < 0.05; nonetheless, for the comparison of disease prevalence rates between sexes, we used the Bonferroni correction method for multiple (18 in this case) comparisons to control familywise error rates, setting statistical significance at *p* < 0.0028. All analyses were performed in STATA (Version 12.0, StataCorp LLC, College Station, TX, USA).

## 3. Results

Between 1 January 2014 and 31 December 2018, 13,062 incident HF patients were identified (mean age of 82 ± 10.5 years, 54.8% women). The main characteristics of the study population are summarised in Table 1. Most patients were aged 85 years or older, followed by those aged 65–84 years. Patients showed a mean number of 4.52 ± 2.06 chronic diseases, although women presented a slightly higher disease burden and multimorbidity prevalence. The prevalence of CV-related and other physical comorbidities was similar between genders, contrary to mental comorbidities, which were more prevalent in women. The most prevalent chronic diseases included AHT, disorders of lipid metabolism, arthritis, and cardiac dysrhythmias, among others. Most of the comorbidities analysed showed a greater prevalence in women, except for cardiac dysrhythmias, DM, AMI, COPD, and neoplasms, which were more prevalent in men. Approximately 67% of the population was polymedicated, with a mean number of 6.75 ± 5 drugs dispensed. The most frequently dispensed drugs were diuretics (59.5%), followed by beta-blockers (34.0%) and angiotensin-converting enzyme inhibitors (ACE inhibitors) and angiotensin II receptor antagonists (ARBs) (30.0%). No significant gender differences were observed, except for the use of beta-blockers, which were more frequent in men, and aldosterone antagonists, which were more frequent in women. Approximately half of the cases of HF were diagnosed during a hospital admission, and one in four died during the one-year follow-up. After HF diagnosis, men showed a greater number of all-cause hospital admissions and readmissions than women (38.4% vs. 33.0% and 17.3% vs. 13.8%, respectively).

The effect of the presence of each group of comorbidities on the five analysed health outcomes in men and women is presented in Table 2. Mental and other physical comorbidities had a greater impact on health outcomes than CV-related comorbidities. HF patients with CV-related diseases showed a 25–27% lower risk of all-cause mortality, with no negative effects on any health outcome analysed. Patients with other physical comorbidities had a 15% lower risk of all-cause mortality, but men showed a 27% higher risk of hospital readmission and women had a 12% higher risk of emergency room visit. Mental comorbidities increased the risk of all-cause hospital admission in both genders and had a different negative effect on other outcomes depending on sex.

**Figure 1:**
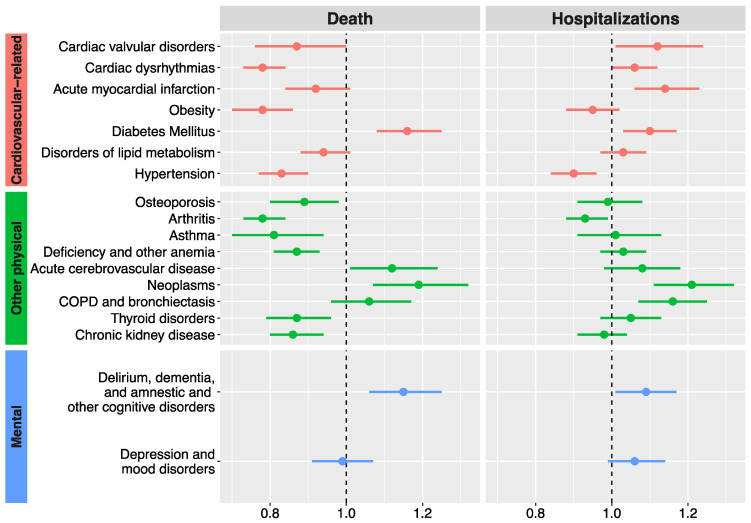
Hazard ratios of mortality and all-cause hospital admission depending on the comorbidities present in patients with heart failure at the moment of diagnosis. COPD: chronic obstructive pulmonary disease. Hazard ratios are adjusted for age and in- or outpatient status at the time of diagnosis and accompanied by their respective 95% confidence intervals.

**Figure 2:**
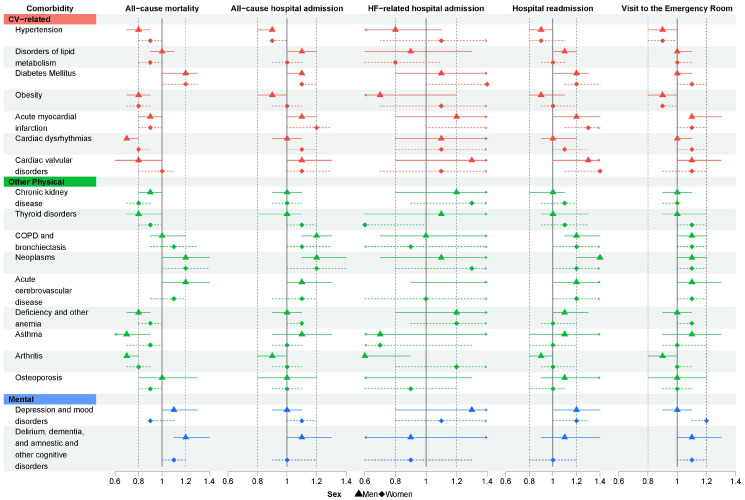
Hazard ratios of the five health outcomes analysed depending on the comorbidities present in patients with heart failure at the moment of diagnosis, stratified by gender. COPD: chronic obstructive pulmonary disease. Hazard ratios are adjusted for age and in- or outpatient status at the time of diagnosis and accompanied by their respective 95% confidence intervals.

The likelihood of all-cause mortality and all-cause hospital admission based on the presence of each particular comorbidity is shown in Figure 1. The effect of each comorbidity on all five health outcomes analysed and stratified by gender is shown in Figure 2, and the specific values of the hazard ratios obtained can be found as supplementary material in Table S1. Neoplasms and DM increased the risk of mortality and hospital admission in both genders. Men with dementia and ACD had a greater risk of mortality, and those with disorders of lipid metabolism, AMI, dementia, and COPD had a higher risk of hospital admission. Women with cardiac dysrhythmias, AMI, and depression and mood disorders seemed to suffer a higher risk of hospital admission. In supplementary Table S2, the results on health outcomes based on each comorbidity group are presented, stratified by age group.

## 4. Discussion

In this study, we analysed the comorbidity of incident HF patients and the effect of the type of comorbidity on several health outcomes and found that, although CV-related comorbidities are the most prevalent and studied conditions in HF patients, it is mental and other physical comorbidities that have a greater effect on mortality and hospitalisation, which also varies depending on gender and the presence of specific comorbidities.

Regarding the characteristics of our cohort of incident HF patients, it is worth highlighting the old age of the individuals as well as the high burden of multimorbidity, which is in accordance with previous studies that reported a high prevalence of comorbidities in HF [2,18,19]. We observed a higher incidence of HF in men, although women were more prevalent in our cohort. This finding might be cohort-specific as other authors did not find differences by gender [18]. Regarding the greater proportion of HF patients living in the most deprived areas, the early manifestation of HF has been widely related to lower socioeconomic status; however, the underlying mechanism has not been clarified [20]. It has been speculated that limited access to healthcare (especially in countries with private healthcare), diet, environmental factors, cardiovascular risk factors and their prevention (smoking, obesity, and AHT), and adherence to treatment may play a role [21].

Data regarding HF-specific pharmacology gave some insight into a crude reality in our population: HF was undertreated and the therapeutic recommendations in force during the study period were poorly followed [14]. The most frequently prescribed drugs were diuretics, but there was scarce use of beta-blockers and ACE inhibitors/ARBs, similar to findings reported in previous studies [22].

The differences observed between men and women in the case of hospitalisations, readmissions, and emergency room visits, which were more frequent in men, could be due to the exacerbations and characteristics of specific comorbidities that are more related to men, as reported in previous studies [23]. Women had a high burden of mental illness, especially depression; however, it was in men that the risk of mortality increased when presenting depression. Several studies have correlated HF with a greater burden of mental conditions. Both depression and cognitive impairment are associated with reduced cerebral blood flow, inflammation, and neurohumoral activations [24], but there are no studies relating it to different mortality rates in men and women. This might be explained by a greater cognitive deterioration or a later diagnosis in men.

Surprisingly, we found that some pathologies grouped as cardiovascular conditions and other physical conditions were associated in both genders with a lower risk of all-cause mortality. These findings could be explained by the fact that conditions that do not share a common pathophysiology with HF can have a greater impact on health outcomes and influence the therapeutic measures adopted, being difficult to manage given the complexity of multimorbid patients [9]. In this sense, HF-concordant pathologies such as the studied cardiovascular conditions would be better treated and have a more intensive follow-up than other comorbidities in HF units [25,26]. The comorbidities most frequently linked to mortality and hospital admission in both genders were predominantly non-cardiovascular, especially neoplasms and mental diseases, apart from type 2 DM, which was indeed one of the comorbidities with a greater impact.

Although there is emerging evidence associating non-cardiac comorbidities with negative health outcomes in HF patients [9,27], studies with larger cohorts are needed to shed some light on the unknown underlying mechanisms. In this sense, inflammation has been pointed out as an important prognostic parameter that appears in both cardiac and non-cardiac conditions. The negative impact on non-cardiac comorbidities has been associated in various studies with a pathophysiological mechanism based on active inflammatory cascades underlying these pathologies and the involvement of advanced HF itself. A low prognostic nutritional index (PNI), which indicates malnutrition and a proinflammatory condition, has been described as a predictor of all-cause mortality during long-term follow-up [28]. This fact could be explained by a higher metabolic rate and a reduction of energy caused by decreased appetite and intestinal absorption, which leads to hypoalbuminemia, which has been related to a high risk of one-year mortality in various published clinical studies [28,29,30]. Several scores, such as MADIT-II, FADES, PACE, and SHOCKED scores, which take into account some of the comorbidities analysed in our study, have been proposed as predictors of mortality in patients with HF. In a study by Hayıroğlu et al. [30], different comorbidities were related to inflammatory markers that, when associated, were considered prognostic values in patients with HF. On the other hand, the fact that most of the CV-related comorbidities were not associated with a higher mortality risk could have been due to the more exhaustive control and treatment of these comorbidities by HF units [25] or maybe to slow progression, meaning that there were undetectable effects during our short follow-up.

In our study, there were more comorbidities associated with hospitalisations than with mortality, especially in the case of men, who usually presented higher rates of hospital admission and readmission compared with women. According to Braunstein et al., there are several reasons that could explain these higher hospitalisation rates in HF patients with a higher comorbidity burden, including the underutilisation of effective HF therapies in the presence of other conditions, a lack of treatment adherence and social support, poor coordination of care, and drug interactions [27]. Another study also found that each additional non-CV mental health disease increased the risk of mortality and hospitalisations in men with HF [9]. In contrast, women had a higher likelihood of visiting the emergency room if they had previous conditions from the other physical or mental comorbidity group.

Although the majority of publications indicate that the most common comorbidities in HF patients are cardiovascular, possibly because they have been the most studied historically, recent studies [9,22] as well as our own study show that comorbidities associated with other physical and mental illnesses are more strongly associated with an increased risk of death and hospitalisation, even after adjusting for confounding factors such as inpatient and/or outpatient status at diagnosis, age, and gender. These findings have important implications for clinical decision-making and the treatment and follow-up of older adults living with HF.

One of the main strengths of our study is its population-based nature and large sample size of incident HF patients diagnosed in both primary and hospital care settings. Additionally, comorbidities were exhaustively studied using different lists of chronic conditions (both cardiovascular and non-cardiovascular), and their impact was analysed using several health outcomes regarding mortality and hospital service use. However, our study also has some limitations. One of the main limitations is the reduced follow-up (one year) of patients, which hindered the assessment of long-term effects on the health outcomes of comorbidities. We decided to exclude data related to the years in which the COVID-19 pandemic took place and extend the recruitment period over five years to increase the number of patients analysed, so we focused on the study of the short-term effect of multimorbidity. On the other hand, the results obtained should be interpreted with caution because of the advanced age of the cohort and the existence of competing risks in these patients, who had in fact more non-HF events than those specifically related to the index disease. Moreover, some relevant clinical variables related to HF prognosis were not available in our cohort, like the left ventricular ejection fraction. Lastly, diagnoses were based on data extracted from electronic health records that were not originally designed for research, leading to potential underestimates of chronic diseases. In this regard, it is worth highlighting that all diagnoses were performed and registered by healthcare professionals from primary and hospital care settings according in all cases to current diagnostic guidelines and recommendations, and that data from the EpiChron Cohort undergo regular quality check-ups and have been the basis of more than 50 scientific publications on multimorbidity.

## 5. Conclusions

Cardiovascular comorbidities are the most commonly described and studied pathologies in HF patients; however, those most strongly associated with a negative impact on health outcomes are non-cardiovascular and mental comorbidities. This fact underlines the need to effectively manage both concordant (i.e., cardiovascular) and discordant (i.e., physical and mental) multimorbidity in the care of HF patients to improve their health outcomes and include recommendations for their proper management in HF clinical practice guidelines.

## Figures and Tables

**Table 1:** Demographic and clinical characterisation of the studied population with incident heart failure.

Characteristics	Men	Women	Total	*p*-Value
**N (%)**	5902 (45.2)	7160 (54.8)	13,062 (100)	
**Mean age, years (SD ^1^)**	80.0 (11.0)	83.6 (9.69)	82.0 (10.5)	<0.001
**Age interval, years (n, %)**				<0.001
≤44	49 (0.83)	44 (0.61)	93 (0.71)	
45–64	489 (8.29)	260 (3.63)	749 (5.73)	
65–84	3007 (50.9)	3021 (42.2)	6028 (46.1)	
≥85	2357 (39.9)	3835 (53.6)	6192 (47.4)	
**Migrant (n, %)**	128 (2.17)	119 (1.66)	247 (1.89)	0.034
**Residence, rural ^2^ (n, %)**	2884 (48.9)	3344 (46.7)	6228 (47.7)	0.014
**Deprivation index ^3^ (n, %)**				0.006
Q_1_	1378 (23.3)	1852 (25.9)	3230 (24.7)	
Q_2_	1415 (24.0)	1673 (23.4)	3088 (23.6)	
Q_3_	1359 (23.0)	1538 (21.5)	2897 (22.2)	
Q_4_	1750 (29.6)	2097 (29.3)	3847 (29.4)	
**Multimorbidity, yes (n, %)**	5424 (91.9)	6818 (95.2)	12,242 (93.7)	<0.001
**Chronic diseases (mean, SD)**	4.21 (1.97)	4.77 (2.09)	4.52 (2.06)	<0.001
**CV comorbidities (%, 95% CI)**	92.4 (91.7–93.1)	93.6 (93.1–94.2)	93.1 (92.6–93.5)	0.006
**Physical comorbidities (%, 95% CI)**	82.3 (81.3–83.2)	87.6 (86.8–88.3)	85.2 (84.6–85.8)	<0.001
**Mental comorbidities (%, 95% CI)**	25.7 (24.6–26.8)	45.5 (44.3–46.6)	36.5 (35.7–37.4)	<0.001
**Prevalence of comorbidities (%, 95% CI)**				
Hypertension	69.3 (68.1–70.4)	77.4 (76.4–78.4)	73.7 (73.0–74.5)	<0.001
Disorders of lipid metabolism	41.5 (40.3–42.8)	44.7 (43.6–45.9)	43.3 (42.4–44.1)	<0.001
Arthritis	38.7 (37.5–40.0)	45.9 (44.8–47.1)	42.7 (41.8–43.5)	<0.001
Cardiac dysrhythmias	39.4 (38.1–40.6)	38.3 (37.2–39.4)	38.8 (38.0–39.6)	0.227
Deficiency and other anaemia	32.9 (31.7–34.1)	38.0 (36.9–39.1)	35.7 (34.9–36.5)	<0.001
Diabetes mellitus	35.6 (34.4–36.8)	29.4 (28.3–30.4)	32.2 (31.4–33.0)	<0.001
Depression and mood disorders	14.6 (13.7–15.5)	31.2 (30.2–32.3)	23.7 (23.0–24.5)	<0.001
Chronic kidney disease	23.6 (22.5–24.6)	23.6 (22.6–24.5)	23.6 (22.8–24.3)	0.993
Obesity	17.2 (16.3–18.2)	20.4 (19.5–21.4)	19.0 (18.3–19.7)	<0.001
Delirium, dementia ^4^	14.1 (13.2–15.0)	22.0 (21.1–23.0)	18.5 (17.8–19.1)	<0.001
Thyroid disorders	9.42 (8.67–10.2)	22.4 (21.5–23.4)	16.6 (15.9–17.2)	<0.001
Acute myocardial infarction	21.5 (20.4–22.6)	12.1 (11.3–12.8)	16.3 (15.7–17.0)	<0.001
Osteoporosis	3.75 (3.26–4.23)	26.0 (25.0–27.0)	16.0 (15.3–16.9)	<0.001
COPD ^5^ and bronchiectasis	24.0 (22.9–25.1)	8.15 (7.52–8.79)	15.3 (14.7–15.9)	<0.001
Neoplasms	13.9 (13.0–14.8)	8.52 (7.87–9.16)	11.0 (10.4–11.5)	<0.001
Acute cerebrovascular disease	10.1 (9.29–10.8)	10.5 (9.77–11.2)	10.3 (9.77–10.8)	0.427
Heart valve disorders	7.34 (6.67–8.00)	7.67 (7.05–8.28)	7.52 (7.06–7.97)	0.475
Asthma	3.96 (3.47–4.46)	10.4 (9.66–11.1)	7.47 (7.02–7.92)	<0.001
**Drugs dispensed ^6^ (mean, SD)**	6.73 (5.02)	6.77 (4.99)	6.75 (5.00)	0.628
**Polypharmacy ^7^, yes (n, %)**	3943 (66.8)	4832 (67.5)	8755 (67.2)	0.411
**Prevalence of drugs of interest (%, 95% CI)**				
Diuretics	60.1 (58.8–61.3)	59.0 (57.9–60.2)	59.5 (58.7–60.4)	0.232
Beta-blockers	35.2 (34.0–36.4)	33.0 (31.9–34.1)	34.0 (33.2–34.8)	0.009
ACE inhibitors/ARBs	30.6 (29.4–31.7)	29.5 (28.4–30.5)	30.0 (29.2–30.7)	0.173
Aldosterone antagonists	21.7 (20.6–22.7)	16.9 (16.0–17.8)	19.1 (18.4–19.7)	<0.001
**Inpatient at HF diagnosis, yes (n, %)**	3305 (56.0)	3671 (51.3)	6976 (53.4)	<0.001
**One-year health outcomes (n, %)**				
All-cause mortality	1531 (25.9)	1785 (24.9)	3316 (25.4)	0.187
Hospital admission related to HF	131 (2.22)	147 (2.05)	278 (2.13)	0.512
All-cause hospital admission	2266 (38.4)	2364 (33.0)	4630 (35.4)	<0.001
Hospital readmission	1020 (17.3)	992 (13.8)	2012 (15.4)	<0.001
Visit to the emergency room	2700 (45.7)	3153 (44.0)	5853 (44.8)	0.050

^1^ Standard deviation. ^2^ Rural versus urban. ^3^ Deprivation index of the residence area according to 26 socio-economic indicators and categorised from least (Q1) to most (Q4) deprived. ^4^ Including amnestic and other cognitive disorders. ^5^ Chronic obstructive pulmonary disease. ^6^ All possible drugs. ^7^ Five or more medications.

**Table 2:** Age-adjusted hazard ratios of 1-year health outcomes depending on the presence of conditions from each comorbidity group in men and women with heart failure. Hazard ratios are accompanied by their respective 95% confidence intervals. Statistically significant hazard ratios *p*-values are highlighted in bold.

	CV-Related Conditions	*p*-Value	Other Physical Conditions	*p*-Value	Mental Conditions	*p*-Value
**Men**						
All-cause mortality	**0.75 (0.63–0.88)**	**0.001**	**0.85 (0.74–0.96)**	**0.012**	**1.21 (1.08–1.34)**	**0.001**
Hospital admission related to HF	1.02 (0.50–2.10)	0.946	1.59 (0.91–2.78)	0.103	1.22 (0.83–1.80)	0.301
All-cause hospital admission	1.02 (0.86–1.21)	0.769	1.06 (0.95–1.19)	0.282	**1.09 (1.00–1.21)**	**0.048**
Hospital readmission	0.98 (0.77–1.26)	0.904	**1.27 (1.06–1.53)**	**0.010**	**1.16 (1.00–1.33)**	**0.041**
Visit to the emergency room	1.01 (0.87–1.18)	0.894	1.10 (0.99–1.23)	0.075	1.08 (0.99–1.18)	0.071	
**Women**						
All-cause mortality	**0.73 (0.62–0.86)**	**<0.001**	**0.85 (0.74–0.96)**	**0.013**	1.01 (0.92–1.11)	0.785
Hospital admission related to HF	0.78 (0.39–1.54)	0.473	1.02 (0.61–1.72)	0.936	0.98 (0.71–1.36)	0.901
All-cause hospital admission	1.07 (0.88–1.29)	0.476	1.09 (0.96–1.25)	0.166	**1.09 (1.01–1.18)**	**0.032**
Hospital readmission	1.19 (0.88–1.63)	0.252	1.13 (0.92–1.39)	0.247	1.11 (0.98–1.26)	0.094
Visit to the emergency room	0.95 (0.81–1.12)	0.551	**1.12 (1.00–1.26)**	**0.047**	**1.15 (1.07–1.23)**	**<0.001**

## Data Availability

The data used in this study cannot be publicly shared because of restrictions imposed by the data owner (i.e., Aragon Health Sciences Institute—IACS) due to the existence of potentially identifying patient information. This restriction has been asserted by the Clinical Research Ethics Committee of Aragón (CEICA). The authors who accessed the data belong to the EpiChron Research Group of IACS and received permission from IACS to utilise the data for this specific study, thus implying its exclusive use by the researchers appearing in the project protocol approved by the CEICA. The EpiChron Group can establish future collaborations with other groups based on the same data. However, each new project based on these data must be previously submitted to the CEICA to obtain the respective mandatory approval. Potential collaborations should be addressed to the Principal Investigator of the EpiChron Research Group, Antonio Gimeno Miguel, at agimenomi.iacs@aragon.es. Requests for the data set used in this study should be addressed to CEICA at ceica@aragon.es.

## Supplementary Materials

The following supporting information can be downloaded at https://www.mdpi.com/article/10.3390/jcm13133979/s1: Table S1: Hazard ratios of all-cause mortality, all-cause hospital admission, HF-related hospital admission, hospital readmission, and visit to the emergency room depending on the presence of each comorbidity in men and women with heart failure. The hazard ratios are adjusted for age and in- or outpatient status at the time of diagnosis and accompanied by their respective 95% confidence intervals. Table S2: Sex-adjusted hazard ratios of all-cause mortality, all-cause hospital admission, HF-related hospital admission, hospital readmission, and visit to the emergency room depending on the type of comorbidity and age group in patients with heart failure. The hazard ratios are accompanied by their respective 95% confidence intervals.
